# Salinity tolerance of three competing rangeland plant species: Studies in hydroponic culture

**DOI:** 10.1002/ece3.3607

**Published:** 2017-11-12

**Authors:** Joseph K. Sagers, Blair L. Waldron, Joseph Earl Creech, Ivan W. Mott, Bruce Bugbee

**Affiliations:** ^1^ Plants, Soils, and Climate Department Utah State University Logan UT USA; ^2^ Forage and Range Research Laboratory USDA Agricultural Research Service Logan UT USA

**Keywords:** dose–response, Gardner's saltbush, halogeton, hydroponics, index: halophyte, kochia, rangeland, salt desert shrub ecosystem, sodium accumulation

## Abstract

Halogeton (*Halogeton glomeratus*) is an invasive species that displaces Gardner's saltbush (*Atriplex gardneri*) on saline rangelands, whereas, forage kochia (*Bassia prostrata*) potentially can rehabilitate these ecosystems. Salinity tolerance has been hypothesized as the predominant factor affecting frequency of these species. This study compared relative salinity tolerance of these species, and tall wheatgrass (*Thinopyrum ponticum*) and alfalfa (*Medicago sativa*). Plants were evaluated in hydroponics, eliminating the confounding effects of drought, for 28 days at 0, 150, 200, 300, 400, 600, and 800 mmol/L NaCl. Survival, growth, and ion accumulation were determined. Alfalfa and tall wheatgrass shoot mass were reduced to 32% of the control at 150 mmol/L. Forage kochia survived to 600 mmol/L, but mass was reduced at all salinity levels. Halogeton and Gardner's saltbush increased or maintained shoot mass up to 400 mmol/L. Furthermore, both actively accumulated sodium in shoots, indicating that Na^+^ was the principle ion in osmotic adjustment, whereas, forage kochia exhibited passive (linear) Na^+^ accumulation as salinity increased. This study confirmed the halophytic nature of these three species, but, moreover, discovered that Gardner's saltbush was as saline tolerant as halogeton, whereas, forage kochia was less tolerant. Therefore, factors other than salinity tolerance drive these species’ differential persistence in saline‐desert ecosystems.

## 1 INTRODUCTION

Gardner's saltbush (*Atriplex gardneri*) is an indigenous perennial shrub in the salt desert shrub ecosystems of the western USA, where it is a valuable source of feed for livestock and wildlife (Smith, Waldron, Creech, Zobell, & Zobell, 2016). Gardner's saltbush has been shown to be vulnerable to invasion from halogeton (*Halogeton glomeratus*), with some land managers reporting complete displacement of the saltbush from halogeton within a time‐span of only 16 years (Goodrich & Zobell, 2011). Furthermore, Smith et al. (2016) reported that the establishment of Gardner's Saltbush proved to be difficult even in its native habitat, especially when a monoculture of halogeton was present.

Halogeton is a fleshy annual weed, native to Eurasia, which was discovered in the United States in 1935 (Dayton, 1951; Young, 2002). Halogeton is a halophyte that reportedly alters the environment in which it lives to obtain a competitive advantage over other plant species (Eckert & Kinsinger, 1960). Soil salts, primarily sodium chloride, are taken up by halogeton roots and transported to the foliage, which is then deposited on the soil surface as leaves and shoots senesce at the end of the growing season. This process, known as “salt pumping,” increases pH, salinity, and exchangeable sodium on the soil surface. The salt persists at the soil surface in arid landscapes where halogeton prevails because there is not enough precipitation to move the salt out of the root zone (Smith et al., 2016). Halogeton has shown optimal growth in the presence of sodium chloride (Cronin & Williams, 1966), which enables it to survive in these altered soils while competing plants cannot (Duda et al., 2003). For livestock producers, this species is of concern as it develops oxalates which are toxic to livestock (Cronin & Williams, 1966).

Forage kochia [*Bassia prostrata* (L.) A.J. Scott; = syn. *Kochia prostrata* L.], a perennial chenopod shrub, is an important forage in its native environment of Eurasia, where it is utilized by sheep, goats, camels, and horses (Waldron, Eun, Zobell, & Olson, 2010). Waldron et al. (2011) recommended the use of forage kochia in western U.S.A., as it is well adapted to these semiarid and arid rangelands and increases nutritional value, carrying capacity, and livestock performance, especially for fall/winter grazing. Forage kochia is reported to have high‐salt and drought tolerance (Francois, 1976; McFarland, Ueckert, Hartmann, & Hons, 1990; Waldron et al., 2010), and has been shown to have potential to rehabilitate disturbed rangeland areas where frequent wildfires occur and invasive annuals such as halogeton displace native perennials (Bailey et al., 2010; Monaco, Waldron, Newhall, & Horton, 2003; Newhall, Monaco, Horton, Harrison, & Page, 2004; Smith et al., 2016).

The high‐salinity tolerance of halogeton, Gardner's saltbush, and forage kochia suggests that these species may be halophytes. Flowers and Colmer (2008) define a halophyte as a plant that can complete its’ life cycle when its natural environment includes salt concentrations of at least 200 mmol/L NaCl. Flowers and Colmer (2008) further defined halophytes as plants that respond positively to NaCl and have optimal growth at the range of 20–500 mmol/L NaCl. Greenway and Munns (1980) separate their classification of halophytes into two different categories: halophytes that grow rapidly at 200–500 mmol/L NaCl, versus those which grow very slowly above 200 mmol/L NaCl. Halophytes are also generally categorized as salt accumulators or salt excluders (Greenway & Munns, 1980). Salt accumulating halophytes often exhibit increased growth as sodium chloride increases, followed by a decrease in growth as salinity approaches toxic levels (Flowers & Colmer, 2008). In contrast, salt excluders, such as many monocot species, have optimum growth in the absence of salt (Flowers & Colmer, 2008).

While salt exists as many different compounds, sodium chloride is the main salt in saline soils that negatively impacts plant growth (Flowers & Colmer, 2008; Glenn, Brown, & Blumwald, 1999; Munns & Tester, 2008). Plant growth is reduced by salt because of both osmotic and specific ion effects on plant cells (Munns & Tester, 2008). The osmotic pressure effect reduces available water at the root zone, which, in turn, causes a loss of water from the cells and a decrease in turgor pressure. Whereas, the uptake of sodium and chloride ions interferes with other internal biochemical processes, causing toxicity (Munns & Tester, 2008). Mechanisms used by plants to tolerate and survive in saline conditions include excluding salt at the root level, limiting transportation to the shoot, moving sodium and excess chloride into the vacuoles, excreting excess salt from the leaves, and accumulation of osmolytes (Glenn et al., 1999; Munns & Tester, 2008). Calcium is an essential element that plants use to preserve structural and functional integrity of cell membranes and cell walls, and to facilitate ion transport and exchange and cell wall enzyme activities (Rengel, 1992), but in saline conditions can be displaced by sodium (Rengel, 1992; Tuna et al., 2007; Volkmar, Hu, & Steppuhn, 1998). Potassium is also an important element in many biochemical and physiological processes within the plant, and under salt stress many plants try to maintain high concentrations of K^+^ in the cytosol (Parida & Das, 2005). Therefore, high levels of K^+^ and Ca^++^ and the ratio between Na^+^ and these ions within the plant are often considered key factors in determining salt tolerance (Volkmar et al., 1998).

The objectives of this study were to: (1) document the comparative salinity tolerance of halogeton, Gardner's saltbush, and forage kochia, and; (2) to determine and/or verify if these species are halophytes by defining their growth and ion accumulation response to increasing levels of salinity. By conducting this trial in a hydroponic environment, comparisons of response to salinity were made between species, without the confounding effect of drought tolerance or limited nutrients. Documenting the relative salinity tolerance helps elucidate the competitive interactions occurring between these species on sensitive and transitional saline rangelands.

## 2 METHODS

### 2.1 Plant materials

The study was conducted in a greenhouse on the campus of Utah State University maintained at 25–27°C during the daytime and 20–25° at night. Entries included in the study were halogeton (*H. glomeratus*; wildland collection), Gardner's saltbush (*A. gardneri*; commercial source variety not stated), alfalfa (*Medicago sativa* subsp. *falcata*; USDA experimental population “HS‐B” selected for salt tolerance), tall wheatgrass (*Thinopyrum ponticum*; USDA experimental population originated from accession PI2555149), gray‐type forage kochia (*B. prostrata* subsp *grisea*; cv “Snowstorm”), and green‐type forage kochia (*B. prostrata* subsp *virescens*; cv “Immigrant”). Entries were started from seed in cone‐tainers filled with 7,030 silica sand and grown for 12 weeks until the juvenile plants reached 10–20 cm in ht. During establishment, they were watered 2× per week by submersing flats of cone‐tainers into a nutrient (Hoagland) solution until cone‐tainers were saturated.

### 2.2 Hydroponics

Following establishment, roots of the juvenile plants were washed, and the plants were placed in hydroponics. Hydroponic tanks, made of high‐density polyethylene, were 175 L in size and were covered with closed cell foam insulation boards. Plant roots were submersed into the hydroponic solution through holes drilled into the foam insulation and soft closed cell foam plugs were used to hold the plants securely in place. The system was aerated by forcing an air supply through PVC pipe with small holes that lay across the bottom of each tank.

The hydroponic solution consisted of 1 g/L nutrient mix (Scotts stock no. 91251/53 Hydro‐Sol), 0.5 g/L of calcium nitrate, 0.15 g/L calcium chloride (dehydrate), and 3 ml/L of 0.1 mol/L potassium silicate mixed with municipal tap water. Calcium nitrate was the plant's main source of nitrogen, whereas, calcium chloride (dehydrate) was added to ensure that ample calcium was supplied. Inasmuch as the purpose of this study was to test the plants ability to monitor osmotic potential, and not necessarily to investigate salinity toxicity, the calcium helped keep sodium levels at low toxicity levels (Greenway & Munns, 1980; Munns, 2002). Silica is not an essential element, but has shown to be beneficial for plant growth especially in hydroponics (Cocker, Evans, & Hodson, 1996; Suriyaprabha, Karunakaran, Yuvakkumar, Rajendran, & Kannan, 2012). Therefore, potassium silicate was added to provide the plants with sufficient silica. The solution pH was maintained at a pH of 5.0 with doses of 0.1 mol/L of nitric acid. In addition, 1 ml of a fungicide (Ridomil Gold EC, active ingredient: Mefenoxam) was added to each tank as a preventative measure. As evapotranspiration occurred, the tank was refilled approximately every 7 days with a modified hydroponic solution. The refill solution consisted of municipal tap water mixed with 0.3 g/L nutrient mix, 0.5 g/L calcium nitrate, and 3 ml/L 0.1 mol/L potassium silicate. These measurements are similar to the original refill solution; however, the nutrient mix was reduced, and calcium chloride was not added because previous experience had indicated that nutrients and calcium are not taken up by the plants at the same rate as evapotranspiration occurred.

### 2.3 Treatments

Treatments consisted of four levels of salinity, and the experiment was arranged in an RCB design with three replications of a single plant, and was repeated three times (runs) with start dates of 15 July, 2015, 30 September 2015, and 9 March 2016. Salinity levels in the first run were 0, 200, 400, and 800 mmol/L of NaCl, and thereafter changed to 0, 150, 300, and 600 mmol/L for runs 3 and 4, due to death of most entries at the 800 mmol/L level. Salinity levels were gradually increased over a period of 10 days until the full molarity was reached in order to minimize plant shock. This was accomplished by each day dissolving in nutrient solution one‐tenth of the total NaCl needed in 175 L and adding it to the respective tanks (153.4, 204.6, 306.8, 409.2, 613.0, and 818.4 g NaCl each day for the 150, 200, 300, 400, 600, and 800 mmol/L treatments, respectively). At the end of 10 days, the solution EC was checked and was always close to the desired ECs of 15, 20, 30, 40, 60, and 80 dS/m for the 150, 200, 300, 400, 600, and 800 mmol/L treatments, respectively. Once final solution molarity was reached the plants were grown an additional 28 days in the hydroponic solution.

### 2.4 Plant growth and element accumulation

Following 28 days of growth in hydroponics at full salinity levels, plant shoots and roots were harvested separately. Shoot and root length were measured following the harvest from the base of the plant to the furthest point on the shoots and the roots. Shoot and root mass were determined by weighing shoots and roots at harvest to determine fresh weight, and then they were dried at 65°C for 72 hr and weighed again to determine dry weight.

Ground shoot samples were sent to the Utah State University Analytical Laboratory (Logan, Utah) for analysis of ion content using a Thermo Electron iCAP ICP (Inductively Coupled Plasma Spectrophotometer) following their standard operating procedure. Root samples were not evaluated. In addition, ground shoot samples were ashed to determine total inorganic content. Ground samples were placed in a microwave ashing oven (Milestone Pyro), and the temperature was raised to 550°C and maintained for 120 min. Following ashing, percent ash on dry matter basis was calculated. Ash‐corrected shoot mass was determined by subtracting the ash content (inorganic content) from the total shoot mass.

### 2.5 Statistical analysis

All data were analyzed with the mixed procedure of SAS to test main effects and get estimates of the Entry × Salinity Level lsmeans and standard errors. Response curves across salinity levels were then fit using Sigmaplot. Shoot and root growth responses were fit to standard dose–response curves using nonlinear three‐parameter sigmoidal logistic model (Equation (1)) as shown:

(1) $$Y=a/\left(1+{\left(\frac{x}{x_{0}}\right)}^{b}\right)$$

where *a* indicates the upper limit, *x*_0_ represents the 50% biomass or growth reduction (e.g., GR_50_) value, *b* is the slope of the line around the GR_50_ values, and *y*_0_ indicates the minimum value obtained. The resulting GR_50_ values provide an objective comparison of salinity tolerance among species. In the case of halogeton, response of shoot mass also required fitting a nonlinear Lorentzian three‐parameter peak model as shown:

(2) $$Y=a/\left(1+{\left(\frac{x-x_{0}}{b}\right)}^{2}\right)$$

where *a* indicates the height of the peak, *x*_0_ represents the location (e.g., salt level) of the peak, and *b* is the scaling parameter which specifies the half‐width at half‐maximum (interquartile range). Shoot ion content response to increasing salinity, in contrast to growth response, was fit using the best available model. In many cases, the best fit for the ion data was sigmoidal, such as the three‐parameter logistic model. However, some species at the higher salinity levels lacked sufficient plant growth for ion analysis, and those responses were mostly fit to a linear polynomial (linear, quadratic, or cubic) model, while a few required nonlinear hyperbola and exponential decay models. The root and shoot growth response models and parameters are listed in Tables 1, 2, 3, 4. For brevity, ion content model parameters are not listed. All growth and ion response fitting analyses were performed on individual plant data.

**Table 1:** Parameter estimates of shoot dry mass in response to increasing salinity levels in a hydroponic study

Entry	Model	*a*	*b*	*x*_0_ a	*R* ^2^
Alfalfa	SL3	55.06 (4.13)	6.86 (7.06)	136.52 (16.99)	0.78
Gardner's saltbush	SL3	15.69 (1.93)	4.27 (3.11)	489.42 (103.90)	0.34
Halogeton	PL3	29.21 (3.29)	243.68 (59.83)	140.67 (35.82)	0.37
Halogeton^b^	SL3	25.20 (3.16)	3.53 (2.17)	463.26 (94.76)	0.33
Immigrant	SL3	55.72 (2.77)	2.29 (0.40)	188.91 (16.21)	0.85
Snowstorm	SL3	45.81 (2.67)	2.18 (0.58)	129.85 (18.67)	0.79
Tall wheatgrass	SL3	29.89 (2.49)	1.54 (1.22)	71.25 (58.98)	0.72

Models used were Sigmoidal Logistic 3 Parameter (SL3), or Peak Lorentzian 3 Parameter (PL3). Standard error stated in parenthesis.

a *x*_0_ is the salt level (mmol/L NaCl) that growth is reduced by 50% (GR_50_) for the logistic model, whereas it is the salt level with highest shoot growth in the Lorentzian peak model.

b Because halogeton had increased growth at low‐salt levels, the Lorentzian peak model is a better fit for the data, but we also forced the logistic model in order to obtain the GR_50_ value.

**Table 2:** Parameter estimates of shoot dry mass as a percent of the control in response to increasing salinity levels in a hydroponic study

Entry	Model	*a*	*b*	*x*_0_ a	*R* ^2^
Alfalfa	SS3	100.01 (4.38)	5.55 (3.19)	130.52 (13.26)	0.91
Gardner's saltbush	SL3	100.71 (9.41)	4.44 (2.70)	531.66 (79.77)	0.39
Halogeton	PL3	143.65 (12.05)	229.02 (39.38)	160.95 (22.88)	0.52
Halogeton^b^	SL3	117.85 (11.57)	4.10 (2.18)	488.96 (75.16)	0.34
Immigrant	SL3	99.69 (2.89)	2.34 (0.23)	197.23 (9.58)	0.94
Snowstorm	SL3	99.97 (4.90)	2.17 (0.48)	132.60 (15.52)	0.84
Tall wheatgrass	SL3	100.00 (4.08)	2.04 (0.60)	105.75 (19.44)	0.91

Models used were Sigmoidal Logistic 3 Parameter (SL3), or Peak Lorentzian 3 Parameter (PL3). Standard error stated in parenthesis.

a *x*_0_ is the salt level (mmol/L NaCl) that growth is reduced by 50% (GR_50_) for the logistic model, whereas it is the salt level with highest shoot growth in the Lorentzian peak model.

b Because halogeton had increased growth at low‐salt levels, the Lorentzian peak model is a better fit for the data, but we also forced the logistic model in order to obtain the GR_50_ value.

**Table 3:** Parameter estimates of shoot dry mass corrected for ash in response to increasing salinity levels in a hydroponic study

Entry	Model	*a*	*b*	*x*_0_ a	*R* ^2^
Alfalfa	SL3	47.45 (4.99)	7.03 (9.27)	138.35 (19.92)	0.69
Gardner's saltbush	SL3	10.51 (1.30)	5.47 (6.64)	532.04 (119.88)	0.26
Halogeton	PL3	18.70 (2.43)	263.60 (81.03)	117.50 (40.62)	0.37
Halogeton^b^	SL3	16.95 (2.11)	3.27 (1.92)	434.87 (89.79)	0.34
Immigrant	SL3	45.36 (2.24)	2.26 (0.41)	185.38 (16.07)	0.85
Snowstorm	SL3	37.01 (2.25)	2.28 (0.70)	130.39 (19.08)	0.79
Tall wheatgrass	SL3	24.63 (2.50)	1.61 (1.87)	78.67 (81.31)	0.67

Models used were Sigmoidal Logistic 3 Parameter (SL3), or Peak Lorentizan 3 Parameter (PL3). Standard error stated in parenthesis.

a *x*_0_ is the salt level (mmol/L NaCl) that growth is reduced by 50% (GR_50_) for the logistic model, whereas it is the salt level with highest shoot growth in the Lorentizan peak model.

b Because halogeton had increased growth at low‐salt levels, the Lorentizan peak model is a better fit for the data, but we also forced the logistic model in order to obtain the GR_50_ value.

**Table 4:** Parameter estimates of root dry mass in response to increasing salinity levels in a hydroponic study

Entry	Model	*a*	*b*	*x*_0_ a	*R* ^2^
Alfalfa	SL3	15.23 (0.61)	2.94 (0.97)	119.61 (15.03)	0.86
Gardner's saltbush	SL3	2.11 (0.19)	1.73 (0.66)	481.13 (99.19)	0.30
Halogeton	SL3	3.55 (0.60)	0.33 (0.70)	66.53 (243.23)	0.12
Immigrant	SL3	9.91 (0.57)	2.58 (0.49)	206.79 (18.32)	0.68
Snowstorm	SL3	7.50 (0.41)	1.29 (0.34)	111.66 (26.44)	0.65
Tall Wheatgrass	SL3	13.99 (0.61)	1.37 (0.49)	74.53 (28.73)	0.82

Model used was Sigmoidal Logistic 3 Parameter (SL3). Standard error stated in parenthesis.

a *x*_0_ is the salt level (mmol/L NaCl) that root growth is reduced by 50% (GR_50_) for the logistic model.

## 3 RESULTS

### 3.1 Plant growth

**Figure 1:**
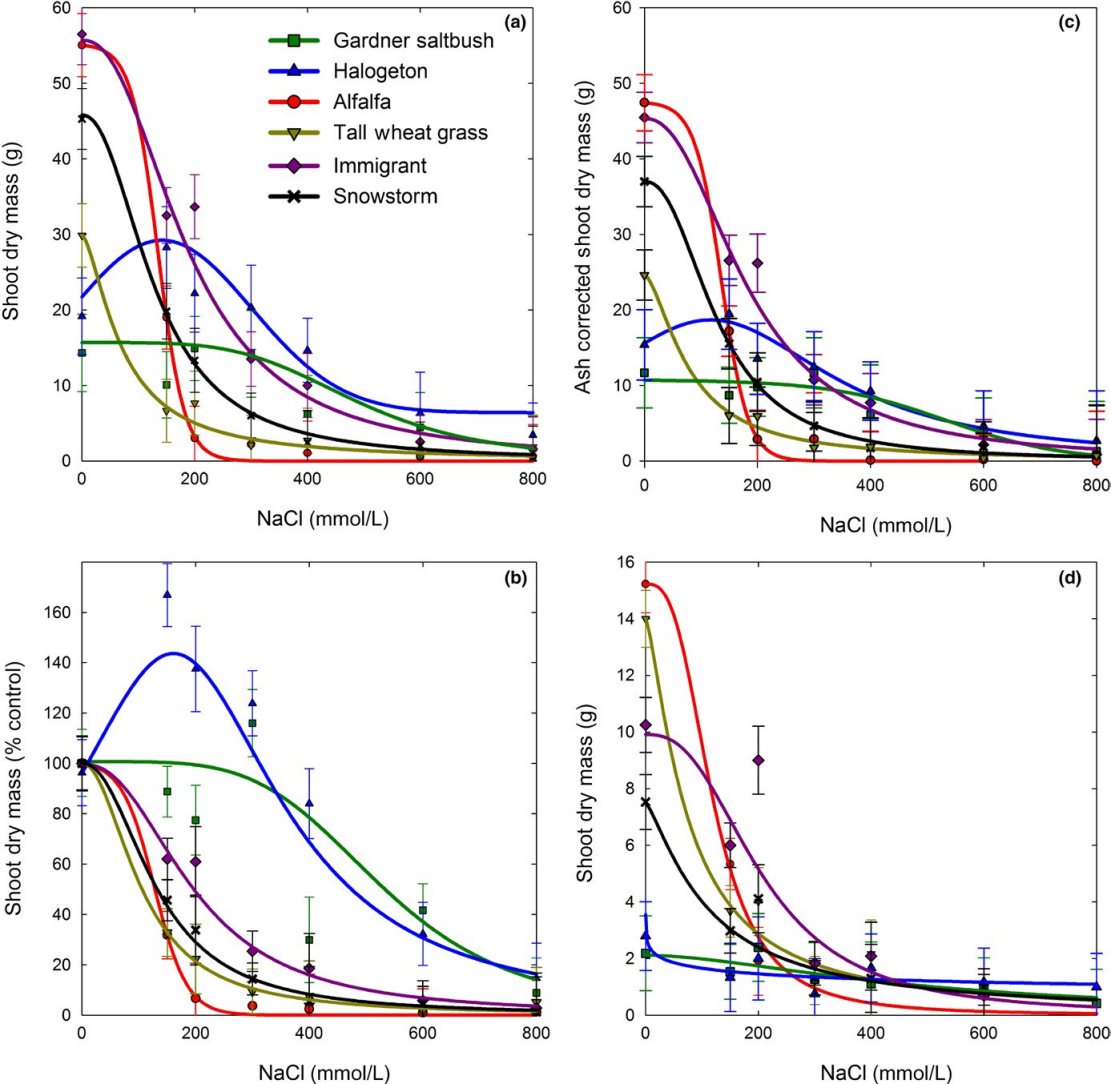
Shoot dry mass (a), shoot dry mass as percent of control (b), ash‐corrected (organic) shoot dry mass (c), and root dry mass (d) of plants grown in hydroponics with increasing amounts of NaCl. Best fit dose–response lines were drawn using parameter estimates shown in Tables 1, 2, 3, 4. Values represent mean ± *SE* (*n* = 6 for 150, 300, and 600 mmol/L, and *n* = 3 for 200, 400, and 800 mmol/L)

Species varied in growth response to increasing salt level, and in general could be categorized into three distinct groups: low‐salt tolerance (alfalfa and tall wheatgrass), medium‐salt tolerance (forage kochia), and highly salt tolerant with obvious halophytic characteristics (Gardner's saltbush and halogeton) (Figure 1). Plant shoot growth in the absence of salt (control) had an inverse pattern, favoring growth of low and medium salt‐tolerant species (Figure 1a). Ash‐corrected shoot mass, as an indication of actual organic growth, was 14%–36% less than total shoot mass, with distinct differences among the species. Averaged across salinity levels, ash‐corrected shoot mass compared to total shoot mass was the most similar for alfalfa (14% less), intermediate for forage kochia and tall wheatgrass (19% less), and the least similar for Gardner saltbush and halogeton (34% and 36% less, respectively) (Figure 1c). However, both ash‐corrected and total shoot mass followed very similar patterns in response to increasing levels of salinity (Figure 1c). Therefore, future references to shoot mass in this publication are of total shoot mass unless otherwise designated.

**Figure 2:**
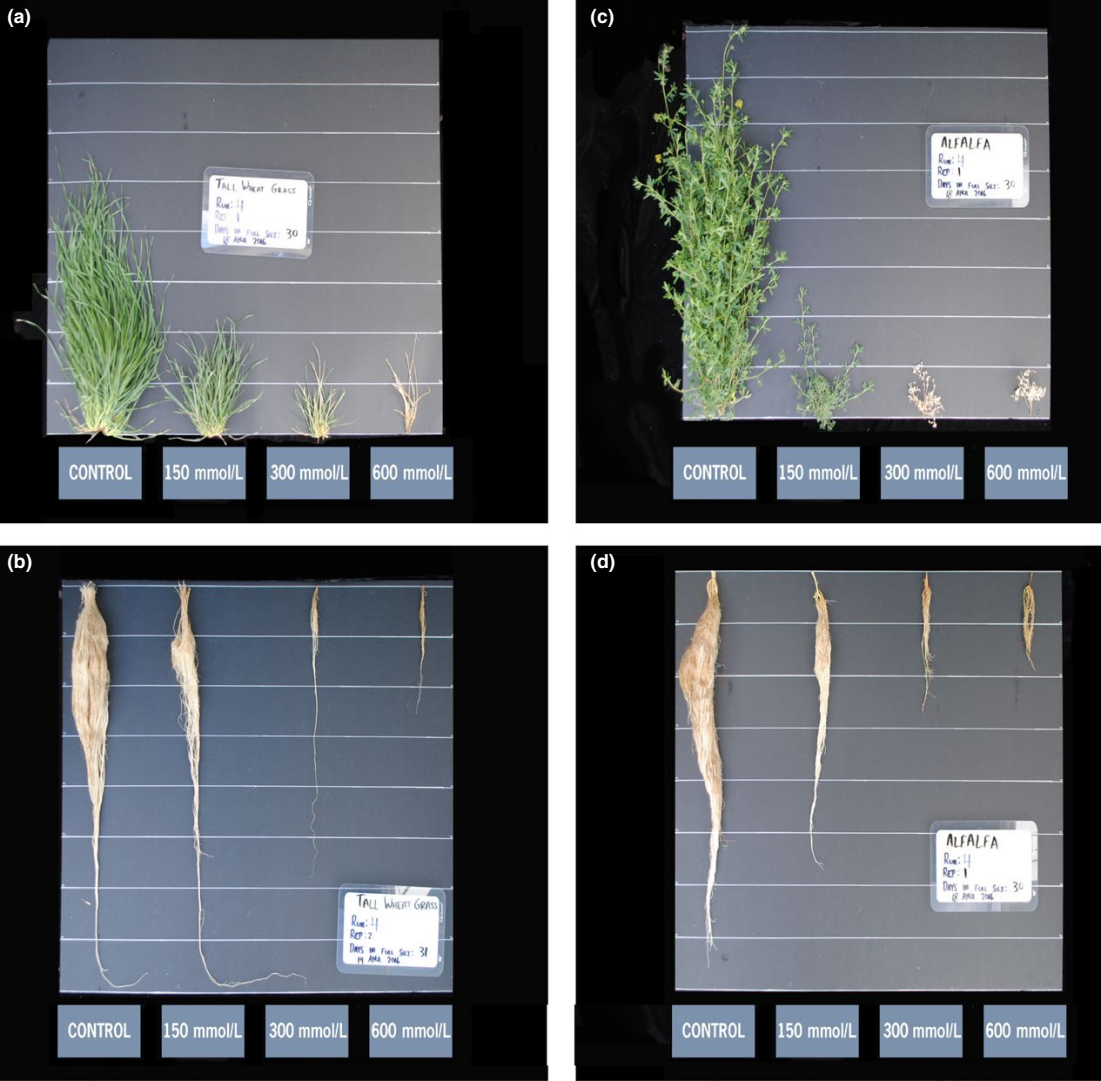
Tall wheatgrass (*Thinopyrum ponticum*) shoots (a) and roots (b); and alfalfa (*Medicago sativa* subsp. *falcata*) shoots (c) and roots (d) after 28 days of growth in hydroponics with increasing amounts of NaCl. Horizontal lines are spaced at 10 cm

Alfalfa and tall wheatgrass were severely affected by increasing salt with both species’ shoot mass reduced to just 32% of the control plants at the lowest salt level (150 mmol/L) (Figure 1b). Interestingly, alfalfa produced greater (*p* = .028) shoot mass (g) than tall wheatgrass at the 150 mmol/L level (Figure 1a), confirming that salt tolerance had been improved in this experimental population of alfalfa. However, tall wheatgrass exhibited overall greater (*p *=* *.0001) salt tolerance than alfalfa, producing low amounts of shoot mass up to the 400 mmol/L level (Figures 1a and 2a). Whereas, alfalfa plants only survived up to the 300 mmol/L level (Figures 1a and 2c), at which point shoot mass amounted to only 3.7% of the control (Figure 1a). Alfalfa and tall wheatgrass produced the most root mass in the absence of salt, and their root mass followed a similar pattern as that of their respective shoot mass, declining most dramatically between the control and the lowest level of salt (Figures 1d and 2b,d).

**Figure 3:**
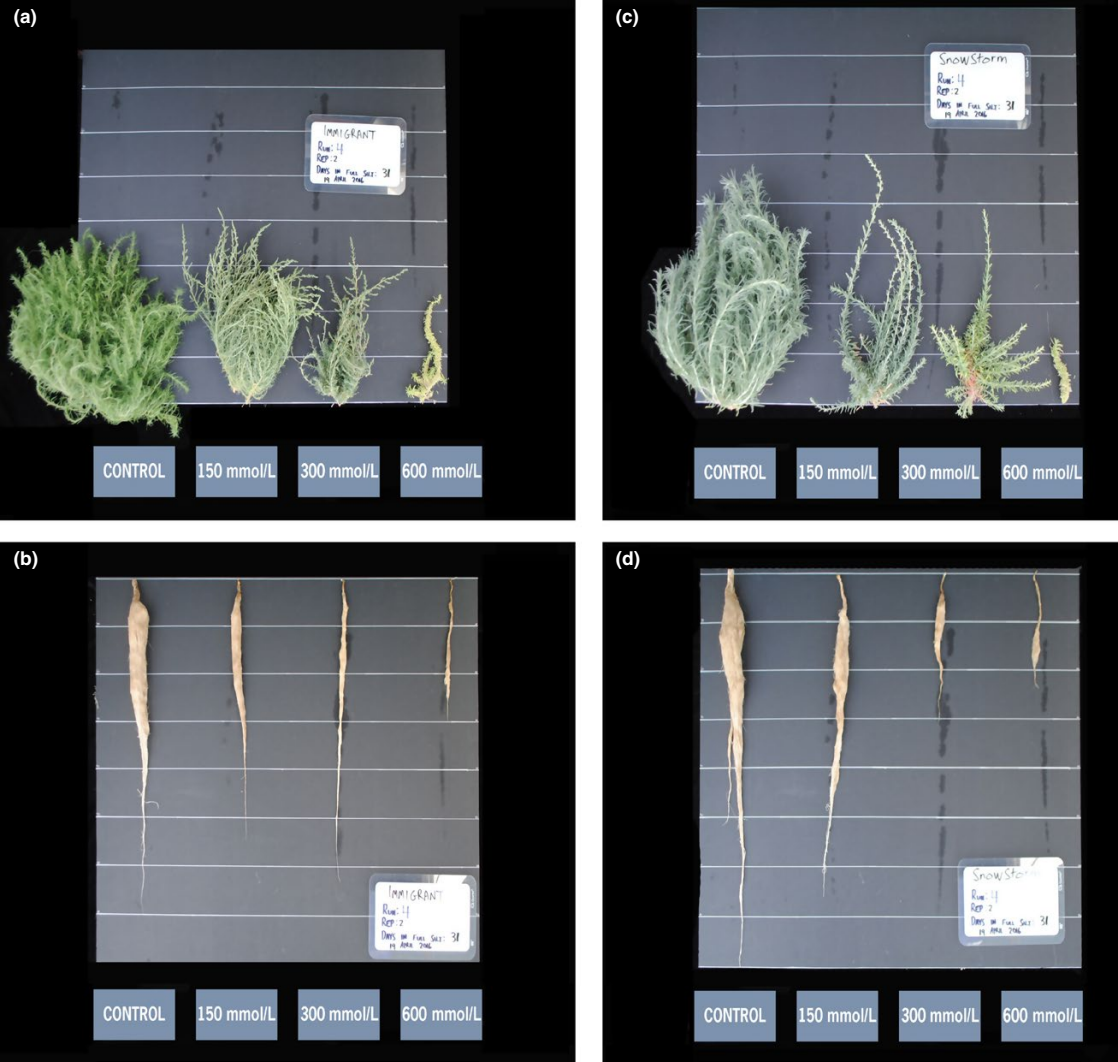
Immigrant forage kochia (*Bassia prostrata* subsp *virescens*) shoots (a) and roots (b); and Snowstorm forage kochia (*B. prostrata* subsp *grisea*) shoots (c) and roots (d) after 28 days of growth in hydroponics with increasing amounts of NaCl. Horizontal lines are spaced at 10 cm

In contrast, the forage kochia entries exhibited greater (*p *=* *.0001–.025) salt tolerance than alfalfa and tall wheatgrass, surviving up to the 600 mmol/L, although they produced little shoot growth at that level (Figures 1a and 3). Forage kochia shoot mass was reduced (*p *=* *.0001) compared to the control even at low‐salt levels, and, thus, they did not exhibit a typical halophytic response of increased growth at low amounts of salts (Figure 1b). Overall, “Immigrant” was more (*p *=* *.0008) salt tolerant than “Snowstorm” with greater shoot mass up to the 400 mmol/L level (Figure 1a). This difference was most pronounced at the 200 mmol/L level (*p *=* *.001), where Immigrant shoot growth was 61% of the control as compared to 34% of the control for Snowstorm (Figure 1b). Immigrant also had greater (*p *=* *.0149) root mass on average than Snowstorm, at the control, 150, and 200 mmol/L salinity levels (*p *=* *.0222, .0077, .0059, respectively) (Figure 1d).

**Figure 4:**
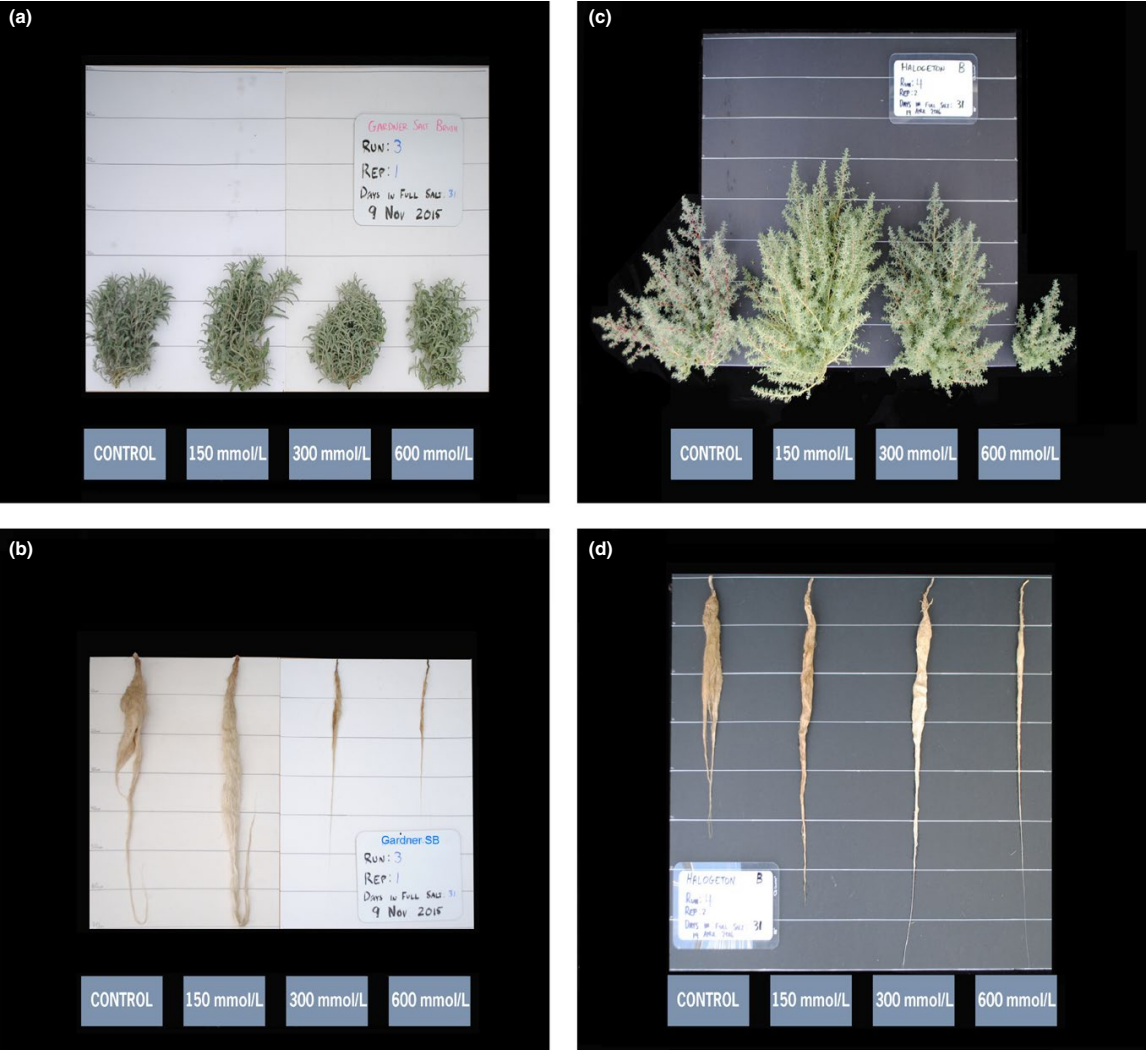
Gardner's saltbush (*Atriplex Gardneri*) shoots (a) and roots (b); and *halogeton* (*Halogeton glomeratus*) shoots (c) and roots (d) after 28 days of growth in hydroponics with increasing amounts of NaCl. Horizontal lines are spaced at 10 cm

Gardner's saltbush and halogeton produced the least overall shoot mass, but their shoot growth indicated that they were the most salt tolerant entries with halophytic‐type growth responses to increasing salinity (Figure 1a,b). Both species had either increasing or stable shoot mass through the lowest salinity levels (Figure 1b), and still produced 15% and 9% of their control's mass, respectively, at the highest 800 mmol/L level (Figures 1b and 4). They also exhibited the least (*p *=* *.05) root mass at 0 mmol/L salinity, but had the most stable root mass across salinity levels, compared to the other species (Figure 1d). Gardner's saltbush root mass never decreased in response to increasing salinity (*p *=* *.5272–.7537), whereas, halogeton root mass was more variable as salinity increased, but never significantly different from the control (*p *=* *.0938–.1705).

### 3.2 Sodium, potassium, calcium, Na^+^/K^+^ and Ca^2+^/K^+^ ratios, magnesium, and phosphorous accumulations

**Figure 5:**
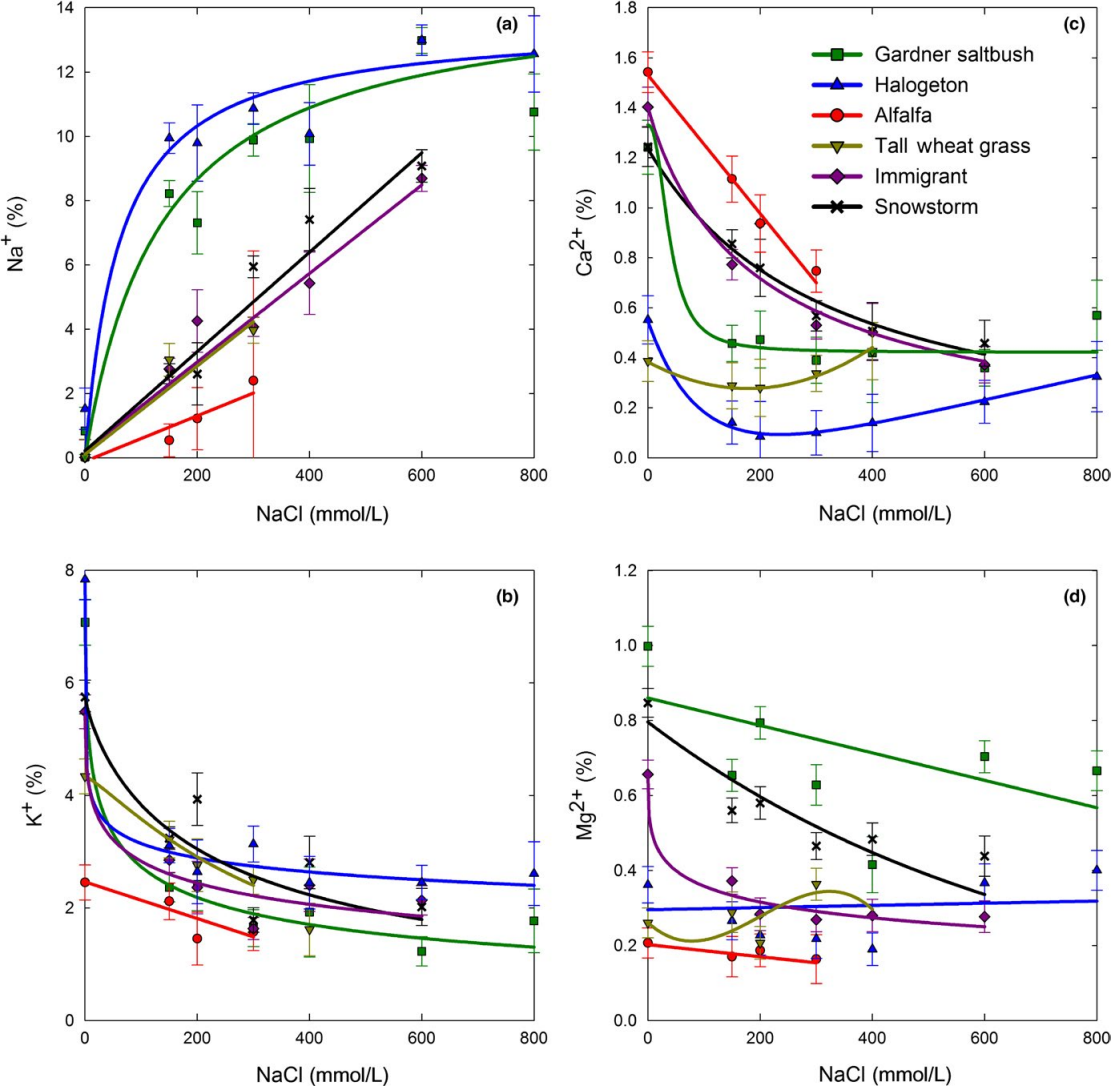
Change in Na^+^ (a), K^+^ (b), Ca^2+^ (c), and Mg^2+^ (d) (% of dry mass) in shoot tissues of plants grown in hydroponics with increasing amounts of NaCl. Best fit dose–response lines are shown. Values represent mean ± *SE* (*n* = 6 for 150, 300, and 600 mmol/L, and *n* = 3 for 200, 400, and 800 mmol/L)

**Figure 6:**
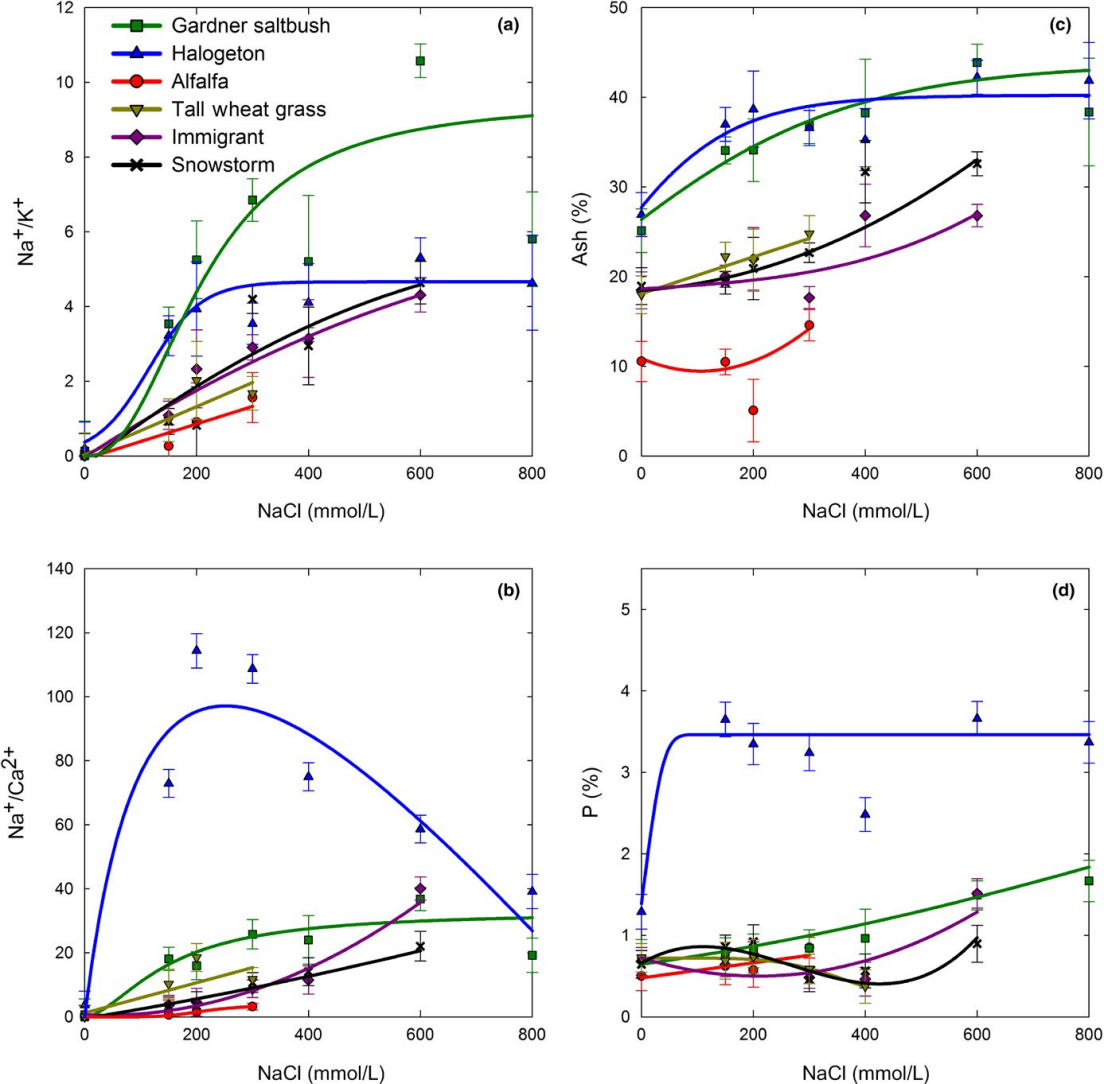
Change in Na^+^/K^+^ (a) and Na^+^/Ca^2+^ (b) ratios, and ash (c) and P (d) content (%) in shoot tissue of plants grown in hydroponics with increasing amounts of NaCl. Best fit dose–response lines are shown. Values represent mean ± *SE* (*n* = 6 for 150, 300, and 600 mmol/L, and *n* = 3 for 200, 400, and 800 mmol/L)

Similar to growth response, Na^+^ accumulation in shoot tissues followed three distinct patterns. Gardner's saltbush and halogeton followed a typical 3‐parameter logistic pattern, where they rapidly accumulated Na^+^ at the 150 mmol/L level (8.2% and 9.9%, respectively), and then gradually leveled off across the higher salinity levels achieving a maximum accumulation of 12.9% of Na^+^ at the 600 mmol/L level (Figure 5a). In contrast, the forage kochia subspecies exhibited a linear increase in Na^+^ accumulation as salinity levels increased, reaching an average of 8.9% at the 600 mmol/L salt level (Figure 5a). The 300 mmol/L level was the highest salinity dose, where alfalfa and tall wheatgrass produced adequate shoot mass to allow for ion analyses. Up to that dose, Na^+^ accumulation in alfalfa was the least of all species (2.4%) and was linearly increasing with greater salinity levels (Figure 5a). In contrast to shoot growth response, tall wheatgrass Na^+^ accumulation more closely resembled that of Immigrant forage kochia than alfalfa, with a maximum of 4.0% Na^+^ at 300 mmol/L salt level (Figure 5a). Potassium content of shoots rapidly decreased in all species as solution salinity increased and Na^+^ accumulated in the shoots (Figure 5b). The decrease in K^+^ was most pronounced in those species that accumulated the greatest amount of Na^+^, reaching their lowest % K^+^ levels at the low‐to‐medium doses of salinity (Figure 5b). Whereas, the decline in K^+^ in tall wheatgrass and alfalfa was linear and more gradual. In comparison, the sodium‐to‐potassium ratio increased linearly with greater salinity in alfalfa, tall wheatgrass, and forage kochia, and as expected, alfalfa had the least Na^+^/K^+^ ratio of all species (Figure 6a). Whereas, Gardner's saltbush and halogeton exhibited a typical logistic dose–response for the Na^+^/K^+^ ratio, and as expected based upon their rate of Na^+^ accumulation, reached maximum Na^+^/K^+^ ratios at medium salinity doses of 300 and 400 mmol/L, respectively (Figure 6a).

In general, Ca^2+^ accumulation in shoot tissues decreased with increasing salinity, with the greatest Na^+^ accumulator (halogeton) exhibiting the lowest Ca^2+^ accumulation (Figure 5c). Interestingly, Ca^2+^ accumulation in halogeton reached its lowest level at 300 mmol/L, increasing marginally thereafter. In addition, the most distinguishable Na^+^/Ca^2+^ ratio response was exhibited by halogeton, with a rapid increase in Na^+^/Ca^2+^ up to the 200 mmol/L level followed by a comparatively rapid decrease as salinity continued to increase (Figure 6b). The greatest Mg^2+^ accumulation occurred in Gardner's saltbush across all salt levels (Figure 5d), whereas, halogeton rapidly accumulated and maintained high levels of P in its shoot tissues in the presence of salinity (Figure 6d).

Ash content has implications to forage nutritive value and is an indicator of inorganic material in tissues. Halogeton and Gardner's saltbush shoots were comprised of large amounts of ash, exceeding 30%, at all salinity levels (Figure 6c). This further indicated that these species rapidly accumulate salt in shoot tissues when grown in saline conditions. The forage kochia entries and tall wheatgrass exhibited intermediate ash content in comparison with other species, and alfalfa had low levels of ash validating that it did not accumulate salt in its shoots (Figure 6c).

## 4 DISCUSSION

### 4.1 Halogeton and Gardner's saltbush's comparative salinity tolerance

Halogeton and Gardner's saltbush have been reported to be salt‐tolerant species, especially in the salt desert shrublands where they commonly grow (Cronin & Williams, 1966; Goodrich & Zobell, 2011). However, this is the first time the salt tolerance of these two species have been compared side by side, in a controlled hydroponic setting that eliminates the confounding effect of drought and limited nutrients. These two salt accumulators were both slow growing, but tolerated salt as high as 800 mmol/L NaCl when grown in this hydroponic system. Halogeton exhibited a typical “halophytic” increase in shoot growth at the lower salinity levels reaching its maximum shoot mass at 141 mmol/L NaCl (Figure 1a; Table 1, *x*_0_ of the Lorentzian model is NaCl level where peak is maximum), and shoot mass was not less than that of the control until salinity reached 400 mmol/L and greater levels (Figure 1b). In a potted plant study, Wang et al. (2015) reported that halogeton reached maximum growth when irrigated with a 100 mmol/L NaCl solution, and declined thereafter with growth at 200 mmol/L significantly less than the control. Wang et al. (2015) irrigated plants daily, but still the differences are probably due to the confounding effect of the variable matrix and osmotic potentials. As water is removed in transpiration, the osmotic potential increases rapidly. This effect is particularly significant in containers because of the reduced root‐zone volume, whereas studies in hydroponic culture minimize this confounding interaction. Wang et al. (2015) also reported that halogeton growth was reduced by 64% at the 500 mmol/L salt level, whereas we found that growth was reduced 50% at a similar salinity (Table 1, 463 mmol/L NaCl is the GR_50_ value). However, even with these slight differences, both studies confirm the high‐salt tolerance of halogeton.

In comparison with halogeton, Gardner's growth response was stable and not affected by salinity up to the 300 mmol/L level (Figure 1a,b). Based upon overall average shoot growth (% of control), halogeton had greater (*p *=* *.0423) salt tolerance than Gardner's saltbush, suggesting support of the hypothesis that halogeton is displacing Gardner's saltbush on rangelands by “salt pumping” to increase soil salinity (Goodrich & Zobell, 2011; Smith et al., 2016). However, examining salinity levels where growth was reduced by 50% (GR_50_) allowed us to directly compare the salinity tolerance of these species. In our study, the GR_50_ values indicate that these two species are more salt tolerant than the other species examined (e.g., 250% greater tolerance than Immigrant forage kochia), and that Gardner's saltbush (GR_50_ = 489 ± 104 mmol/L) and halogeton (GR_50_ = 463 ± 95 mmol/L) have nearly identical salinity tolerance (Table 1). Moreover, ash‐corrected GR_50_ values suggest that Gardner's saltbush (532 ± 120 mmol/L) has greater salt tolerance than halogeton (435 ± 90 mmol/L) (Table 3). Therefore, this study clearly indicates that factors other than salt tolerance, including drought or rhizosphere alteration by halogeton (Duda et al., 2003; Smith et al., 2016), are likely primarily responsible for the displacement of Gardner's saltbush by halogeton.

Both halogeton and Gardner's saltbush accumulated sodium in shoot tissues (Figure 5a). Even at the least dose of 150 mmol/L NaCl, both species had accumulated Na^+^ in shoot tissues that were over 40 time greater than salt concentrations considered toxic to plants (0.2%) (Bernstein, 1975) (Figure 5a). In addition, the Na^+^/K^+^ ratios were at minimum five times greater than optimum for nonhalophytic plant growth (Greenway & Munns, 1980) (Figure 6a). These results suggest that the tolerance mechanism of these halophytic species is primarily osmotic adjustment, associated with the compartmentalization of Na^+^ (Munns & Tester, 2008). This is in agreement with Wang et al. (2015) who reported that halogeton salt tolerance came from osmotic adjustment associated with transport and compartmentalization of sodium in vacuoles. They reported a Na^+^ content of 17% of dry weight in halogeton leaves at 500 mmol/L NaCl level, whereas in our study, sodium content at 600 mmol/L NaCl was 12% of dry weight for both halogeton and Gardner's saltbush (Figure 5a). The difference may be because we measured the sodium content of the entire shoot, which suggests that the stems also compartmentalized Na^+^, but not to the same level as the leaves. Our data show that Na^+^ was the principle ion involved in osmotic adjustment in both of these species, with Na^+^ accumulation (Figure 5a) resembling that observed for active uptake of essential nutrients resulting in concentrations higher in the plant than that in the external environment (White, 2012). In addition, their ability to transport Na^+^ into the shoot appeared to be saturated at relatively low external salinity, similar to that observed for *Suaeda maritima* (Yeo & Flowers, 1986), a succulent halophyte like halogeton, and *Atriplex canescens*, another common *Atriplex* shrub species found on salt desert shrublands of North America (Glenn, Olsen, Frye, Moore, & Miyamoto, 1994). In comparisons of Gardner's saltbush to four‐wing saltbush (*A. canescens* subsp. *canescens*), Gardner's saltbush accumulated greater amounts of Na^+^ and had greater Na^+^/K^+^ ratios in high saline environments than did four‐wing saltbush (Glenn, Pfister, Brown, Thompson, & O'Leary, 1996; Glenn, Watson, O'Leary, & Axelson, 1992; Glenn et al., 1994). However, Glenn et al. (1992) concluded that high‐salt tolerance in *A. canescens* was not completely dependent upon high levels of Na^+^ accumulation.

Ash content, as a measure of inorganic material in the shoots, provided further evidence of the high‐sodium uptake and accumulation in halogeton and Gardner's saltbush (Figure 6c). In this study, halogeton and Gardner's saltbush had ash contents ranging from 37% to 42% and 34% to 44%, respectively, for salinity levels ranging from 150 to 600 mmol/L (Figure 6c). These extreme values exceed those previously reported for Gardner's saltbush (25% ash) when sampled from plants growing in its natural salt desert shrub rangeland environment (Welch, 1978). Most other nutrient and ion concentration trends in halogeton and Gardner's saltbush were as expected with sodium accumulators. In general, as these species increased uptake of sodium, there was an associated decrease in uptake of K^+^, Ca^2+^, and Mg^2+^ (Figure 5b–d). The response was rapid, occurring mostly by 200 mmol/L NaCl, except in the case of Mg^2+^ where a gradual decrease was observed in Gardner's saltbush and no decrease was exhibited by halogeton as salinity increased. Phosphorus uptake by halogeton was also noteworthy (Figure 6d). Halogeton plants at all salinity levels accumulated phosphorous such that shoot concentrations exceeded 10 times that considered adequate for a growing plant (0.3%–0.4%).

### 4.2 Is *Bassia prostrata* a halophytic species?

Forage kochia is considered a drought and salt‐tolerant species (Waldron et al., 2010), and, in preliminary studies, it exhibited high‐salt tolerance including active growth and LD_50_ values at salinity levels exceeding that of seawater (600 mmol/L NaCl) (unpublished data). However, in those studies, more mature forage kochia plants and/or potted plant experiments were used, and they were not compared to a documented halophyte such as halogeton. This is the first known report of forage kochia's salinity tolerance without the confounding effect of drought tolerance.

Unlike that observed for halogeton and Gardner's saltbush, shoot mass of forage kochia decreased at even the lowest salt level of 150 mmol/L (Figure 1a,b). Karimi, Ghorbanli, Heidari, Khavari Nejad, and Assareh (2005) reported that forage kochia growth was not decreased at salinity levels between 50 and 150 mmol/L, and then exhibited a 52% shoot reduction at 200 mmol/L NaCl. Our study was similar to theirs with the same initial size and age of forage kochia seedlings, the same rate of incremental increase to reach full salinity (10% increase in salinity each day for 10 days), and the same duration of the study, but the primary differences were that they used plants potted in sand and examined responses at salinity levels below 150 mmol/L. Normally, due to evapotranspiration, potted plants would have higher root‐zone salinity than the actual solution salinity. Our study did not look at salinity below 150 mmol/L so we cannot directly compare to their results at 50 and 100 mmol/L, but similar results might have been obtained or even increased growth at those lower levels. Additionally, genetic differences between populations may be responsible for the differences detected between our two studies. Their plants originated from wildland collected seed in Iran (Karimi et al., 2005) that were likely indigenous to saline environments; whereas, Immigrant germplasm originates from an unknown location in Russia (Stevens, Jorgensen, McArthur, & Davis, 1985) and Snowstorm originates from germplasm sources in Uzbekistan (Waldron et al., 2013). While this species is noted for its salt tolerance (Francois, 1976; Waldron et al., 2010), neither of these cultivars was purposely selected for salt tolerance, and both are many generations removed from their original habitat. However, even so our calculated GR_50_ of Immigrant (189 mmol/L) (Table 1) is in the same general range of that observed for the Iranian biotype (between 150 and 200 mmol/L).

Halophytes often accumulate sodium in shoot tissues as a mechanism for osmotic potential adjustment (Flowers & Colmer, 2008). In contrast to the active uptake observed for halogeton and Gardner's saltbush, forage kochia exhibited passive uptake of Na^+^ as evidenced by a linear increase in sodium content of shoots as salinity increased (White, 2012) (Figure 5a). Karimi et al. (2005) also observed a linear increase in shoot sodium content in forage kochia as salinity increased from 0 to 200 mmol/L. However, their sodium accumulation was double (5.5% of shoot dry matter) of that which we observed (2.7%) at the 150 mmol/L salt level. The fact that their control plants contained 1.2% sodium in the shoots as opposed to our range of 0.1%–0.3% in forage kochia control plants, suggests the possibility of their control solution containing higher sodium than ours and may be one reason some results differ. In addition, Karimi et al. (2005) reported 50% less K^+^ accumulation and nearly triple Na^+^/K^+^ of that we observed, further indicating that there were likely differences in experimental solutions and overall conditions. They conclude that *B. prostrata* is a halophytic species with optimum growth at 150 mmol/L NaCl, and maintains osmotic potential by NaCl accumulation in vacuoles. Even though we observed a substantial growth decrease at the 150 mmol/L salinity level, our findings support their conclusion that forage kochia is a halophyte as many other indicators were in common including sodium accumulation in the shoot tissues. In addition, our study examined much higher salt levels, and we found that even though growth was severely reduced, *B. prostrata* plants survived up to the 600 mmol/L salt level (Figure 3), further supporting its classification as a halophytic species.

The salt tolerance of Snowstorm forage kochia was less than that of Immigrant (GR_50_ values of 130 and 189, respectively) (Table 1). Smith et al. (2016) reported that Immigrant performed better than Snowstorm in a halogeton‐invaded Gardner's saltbush ecosystem. They were surprised by this finding inasmuch as they had surmised that Snowstorm and the subsp. *grisea* had greater salt tolerance than Immigrant and the subsp. *virescens*. Our results do not support their expectations concerning the relative salt tolerance between these two forage kochia subspecies, and provide additional evidence that Immigrant was better adapted than Snowstorm to their saline, halogeton‐invaded, test environment.

### 4.3 Conclusions about comparative salt tolerance

Based upon GR_50_ values for shoot mass (Tables 1, 2, 3), the salt tolerance of these species would be ranked in this order: Gardner's saltbush = halogeton > forage kochia (Immigrant > Snowstorm)  > alfalfa > tall wheatgrass. It is remarkable that alfalfa would be reported to have greater salt tolerance than tall wheatgrass, and, based upon these measurements, it was also equal in salt tolerance to Snowstorm forage kochia. In this study, we used a salt‐tolerant experimental alfalfa population (HS‐B) that in an earlier study exhibited greater salt tolerance than the parent population at the 90 mmol/L salinity level (Anower, Mott, Peel, & Wu, 2013). However, our salt levels were higher than those examined by Anower et al. (2013), and, in our study, HS‐B had the least shoot biomass at all salt levels above 150 mmol/L. It is probable that a comparison of these entries at salt levels ranging between 0 and 150 mmol/L would give a more accurate estimate of GR_50_ and change the salt tolerance ranking between alfalfa, tall wheatgrass, and Snowstorm forage kochia. Nevertheless, our results support their findings that this alfalfa germplasm has been selected for improved salt tolerance and that the salt tolerance mechanisms for HS‐B include excluding sodium transport to the shoots. However, at salinity levels greater than what they evaluated (e.g., >90 mmol/L NaCl), some sodium accumulated in the shoots of this alfalfa population (Figure 5a). Tall wheatgrass has been characterized as both a salt tolerant and a halophytic grass (Shannon, 1978). In our study, it was the least salt‐tolerant species (based upon GR_50_ values), but accumulated sodium in a similar pattern and rate (passive accumulation) as forage kochia (Figure 5a) until Na^+^ levels apparently reached toxicity, as evidenced by plant death (Figure 2a) at salinity of 400 mmol/L and greater. Further evidence of halophytic growth in tall wheatgrass was a Na^+^/K^+^ ratio that was intermediate between forage kochia and alfalfa and above what expected for a nonhalophyte (<0.6) (Greenway & Munns, 1980) at salinity levels ranging from 150 to 300 mmol/L (Figure 6a).

## 5 CONCLUSIONS

This study evaluated the comparative salt tolerance of several putative halophytic plant species, and confirmed that halogeton is a halophytic species, and, thus, it has an adaptive advantage on the salt desert shrublands of North America. The salt tolerance of the *Atriplex* genus (saltbushes) has been widely examined, and our data indicate that Gardner's saltbush is yet another *Atriplex* species with halophytic properties. We have documented that Gardner's saltbush is equally as salt tolerant as halogeton, suggesting that growth and other competitive factors are responsible for the displacement of Gardner's saltbush by invasion of halogeton. Furthermore, we confirmed that although *B. prostrata* (forage kochia) is a halophytic species capable of survival at salinity levels equal to seawater, it does not have as great of salt tolerance (as determined by GR_50_) as Gardner's saltbush and halogeton. Inasmuch as researchers have reported the potential for forage kochia to rehabilitate halogeton‐invaded Gardner's saltbush ecosystems, this further indicates other traits such as drought tolerance are important for plant survival and competition on these saline rangelands. Additional hydroponic studies examining salinity levels below 150 mmol/L, and possible using older plants and a broader range of genotypes could further elucidate salinity tolerance of forage kochia.

## CONFLICT OF INTEREST

None declared.

## AUTHOR CONTRIBUTIONS

J.K. Sagers contributed to the design, acquired the data, and drafted the article. B.L. Waldron made substantial contributions to conception and design, oversaw data acquisition, completed analysis and interpretation of data, revised the article, and gave final approval. J.E. Creech made substantial contributions to conception and design, review and revision of the article, and gave final approval. I.W. Mott made substantial contributions to conception and design, review and revision of the article, and gave final approval. B.B. Bugbee made substantial contributions to conception and design, helped oversee data acquisition, contributed to review and revision of the article, and gave final approval.
